# The “Ultimate Prize” for Big Tobacco: Opening the Chinese Cigarette Market by Cigarette Smuggling

**DOI:** 10.1371/journal.pmed.0030279

**Published:** 2006-07-18

**Authors:** Thomas E Novotny

## Abstract

Novotny discusses a new study in *PLoS Medicine* that documents how British American Tobacco has exploited China's large cigarette smuggling problem.

## The Prize

China has long been identified as the most important international cigarette market, now and in the future—O'Sullivan and Chapman called China the “ultimate prize” among the world's emerging tobacco markets [ 1]. Almost two-thirds of Chinese men (63%), and 3.8% of Chinese women, are smokers [ 2], giving a total of 350 million smokers in China, with more on the way. Young Chinese women are likely to be an important target group for growth for Big Tobacco.

According to a 1998 survey, among Chinese adolescents aged 11–20 years, almost half (47.8%) of the boys and 12.8% of the girls surveyed were experimenting with tobacco [ 3]. This survey would surely warm the cold heart of any multinational tobacco company executive. Robert Fletcher, regional public affairs manager for the tobacco company Rothman, said that “thinking about Chinese smoking statistics is like trying to think about the limits of space” [ 4].

China has been a member of the World Trade Organization since 2001, and this membership may reduce barriers to market entry through lowered tariffs on imports by transnational tobacco companies (TTCs) [ 5]. Thus, it is important to understand how these markets will be entered by the TTCs, as market entry also means globalizing the imagery, advertising, and health effects of smoking [ 6]. Market entry into China also means that with greatly increased numbers of smokers, the burden of non-communicable diseases attributable to smoking will explode, affecting China's transitional health-care system and its economic stability.

## Smuggling as a Marketing Tool

TTCs have used cigarette smuggling as a market-opening tool throughout recent history (such as in Latin America [ 7] and the former Soviet Union [ 8]). Now, in an important new study published in *PLoS Medicine* [ 9], Lee and Collin document evidence that British American Tobacco (BAT) has exploited China's large cigarette smuggling problem.

Smuggling of cigarettes into China is an especially critical issue in light of China's desire both to open trade through membership in the World Trade Organization and to participate more fully as a global partner in health. With the 2002 SARS epidemic and the potential avian flu pandemic serving as grim reminders of the need for international collaboration in health, China now participates more openly in other health agreements. Specifically, China ratified the World Health Organization's first ever global health treaty, the Framework Convention on Tobacco Control (FCTC) [ 10], on October 11, 2005. This treaty will have as one of its major areas of focus the control of global cigarette smuggling [ 11]. Although the United States has not ratified the FCTC, it may join in a protocol that addresses smuggling. At present, one-quarter of total global cigarette exports are illegally traded. This illegal trade is a big, multinational problem, and multinational efforts are needed to control it; in addition, there is still insufficient scholarly research on this problem [ 12].

“Thinking about Chinese smoking statistics is like trying to think about the limits of space.”

## Getting Inside Information

Lee and Collin used BAT documents from the Guildford Depository in the United Kingdom. These documents were made public as a result of legal actions against the TTCs. The terms of a legal settlement in Minnesota, United States, stipulated that the public should be allowed, for ten years, access to documents produced during litigation against the tobacco industry, via the creation of two depositories, one in Minnesota and one in Guildford [ 13].

Document research is of specific value in examining the smuggling issue, since data on smuggling are not easily found in other public sources. The authors found suggestions of large-scale smuggling of foreign cigarettes into China to circumvent barriers to market access. By triangulating official documents on legal imports subject to tariffs, reported imports by all TTCs, and BAT sales figures in China, they found significant discrepancies between what was reported and what was sold. The sales reported in internal company documents made China one of the larger profit centers for BAT Industries, with 25% of profits coming from “transit” trade to China, a code word for smuggled cigarettes. Moreover, the Guildford documents show evidence of BAT's efforts to build market presence in competition with other popular brands, using whatever it takes to succeed in the new market, including smuggling.

## What Must Be Done

The FCTC came into force on February 27, 2005. The signatory member states now have the best-ever opportunity to shut down the pipeline of smuggled tobacco products and the associated illicit profits for BAT and other TTCs through multinational efforts to better track exports and enforce customs regulations. There are three major reasons why it is crucial to stop tobacco smuggling: (1) the profits from smuggling fuel TTCs' efforts to buy political influence and maintain economic strength across non-tobacco sectors; (2) smuggling leads to countries losing tariff revenue from the legal trade of cigarettes; and, most importantly, (3) the health of target groups such as young Chinese women will suffer immeasurably unless the FCTC is effective in reducing TTC market success.

Much can be done to combat smuggling as a tool of the TTCs, including implementing more aggressive litigation to assure corporate liability, pursuing anti-money-laundering actions to remove the influence of organized crime [ 14], improving cigarette tracking systems, and strictly enforcing record-keeping of shipments and receipts by TTCs. There must be binding obligations to carry out such anti-smuggling activities, enforced through a multinational protocol of the FCTC, not voluntary compliance as proposed by the TTCs. The world is a global marketplace, and global problems such as cigarette smuggling need global solutions as put forth in the FCTC.

## Abbreviations

BAT: British American Tobacco
FCTC: Framework Convention on Tobacco Control
TTC: transnational tobacco company
